# Serial block‐face scanning electron microscopy reveals novel intercellular connections in human term placental microvasculature

**DOI:** 10.1111/joa.13191

**Published:** 2020-04-03

**Authors:** Eleni Palaiologou, Patricia Goggin, David S. Chatelet, Rodolfo Ribeiro de Souza, Wendy Chiu, Brogan Ashley, Emma M. Lofthouse, Bram G. Sengers, Christopher Torrens, Anton M. Page, Jane K. Cleal, Rohan M. Lewis

**Affiliations:** ^1^ Human Development and Health Faculty of Medicine University of Southampton Southampton UK; ^2^ Biomedical Imaging Unit Faculty of Medicine University of Southampton Southampton UK; ^3^ Faculty of Engineering and Physical Sciences University of Southampton Southampton UK; ^4^ Institute for Life Sciences University of Southampton Southampton UK

**Keywords:** endothelial junction, filopodia, microvasculature, placenta, villi

## Abstract

The placental microvasculature is a conduit for fetal blood allowing solute exchange between the mother and the fetus. Serial block‐face scanning electron microscopy (SBF SEM) allows ultrastructure to be viewed in three dimensions and provides a new perspective on placental anatomy. This study used SBF SEM to study endothelial cells within the human placental microvasculature from uncomplicated pregnancies. Term human placental villi were aldehyde‐fixed and processed for imaging by SBF SEM. Manual segmentation was carried out on a terminal villous capillary and an intermediate villous arteriole and venule. Twenty‐seven SBF SEM stacks from terminal villi were analysed using stereological approaches to determine the volumes of microvascular components and the proportions of pericyte coverage. SBF SEM analysis of capillary endothelial cells revealed the presence of interendothelial protrusions (IEPs) originating from the donor cell at the endothelial junction and forming deep thin projections up to 7 μm into the adjacent endothelial cells. IEP density was estimated to be in the order of 35 million cm^–3^ placental tissue. Pericytes cover 15% of the fetal capillary surface area in terminal villi. In comparison, the cytotrophoblast covered 24% of the syncytiotrophoblast basal membrane. A trans‐endothelial channel was observed in a region of the vasculo‐syncytial capillary. Pericyte coverage was extensive in both arteriole and venule. Three‐dimensional imaging of the placental microvasculature identified novel ultrastructural features and provided an insight into factors that may influence capillary permeability and placental function. We hypothesise that the IEPs may allow mechanosensing between adjacent endothelial cells to assist in the maintenance of vessel integrity. The numbers of endothelial junctions, the presence of trans‐endothelial channels and the extent of pericyte coverage all provide an insight into the factors determining capillary permeability.

## Graphical abstract

Serial block‐face scanning electron microscopy to create a 3D reconstruction of endothelial cells. Interendothelial protrusions were observed originating from a donor endothelial cell and tunnelling inside a recipient endothelial cell. These structures constitute a novel intercellular connection.

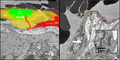

## 1 INTRODUCTION

The placenta mediates the exchange of gases, nutrients and waste products between the maternal and fetal blood. Placental function is required to support fetal development which establishes the foundations for health across the life course (Lewis *et al.*, 2012). Three‐dimensional imaging approaches are allowing new insights into placental structure as well as the exploration of structure‐function relationships using computational modelling (Plitman Mayo *et al.*, 2016; Perazzolo *et al.*, 2017; Palaiologou *et al.*, 2020).

The placental microvasculature mediates the flow of fetal blood to the terminal villi where exchange occurs and must also maintain an appropriate level of vascular permeability to allow the exchange of nutrients and wastes. The placental microvasculature includes the arterioles and venules within intermediate villi and the capillaries within terminal villi (Leach and Firth, 1997). Within the villi, blood flows through arteries and veins in stem villi, arterioles and venules in intermediate villi and capillaries in terminal villi. The organisation of vessels within the human placenta means that all the blood that flows up arterial vessels of a branch of the villous tree must flow back down the venous vessels of the same branch. Terminal villi contain specialised vasculo‐syncytial regions where the capillaries are dilated and lie in close contact with the placental barrier: the syncytiotrophoblast. Most nutrient exchange is thought to occur in these vasculo‐syncytial regions where diffusion distance is shortest (Burton and Tham, 1992). While the syncytiotrophoblast is the primary barrier to exchange, the trophoblast basal lamina, villous stoma and the fetal capillary endothelium are additional barriers to transfer. In human placenta, the arterioles and venules of intermediate villi do not have smooth muscle but are surrounded by pericytes, which are also associated with villous capillaries.

Endothelial cells are not static structures and when cultured undergo dynamic remodelling (Guo *et al.*, 2007). The integrity of endothelial junctions and the localisation of junctional complexes reflect physical forces on the cells (Valent *et al.*, 2016). Effective communication between endothelial cells is likely to be essential in maintaining vascular integrity.

The permeability of the endothelial junctions in the placenta is estimated to be similar to that in skeletal muscle (Leach and Firth, 1997). The passive permeability of placental capillaries will be determined by the presence of transcellular routes, including endothelial junctions and trans‐endothelial channels (Wagner *et al.*, 2012). Permeability of endothelial junctions will be determined by the tight and adherens junctional complexes and by the extent to which pericytes cover the endothelial junctions (Bazzoni and Dejana, 2004).

Endothelial cells have been studied extensively using two‐dimensional microscopy approaches but new techniques such as serial block‐face scanning electron microscopy (SBF SEM) allows a greater understanding of placental ultrastructures in three dimensions (Denk and Horstmann, 2004). This technique uses a scanning electron microscope with an ultramicrotome inside to generate serial images of a resin embedded sample. The resin block is cut to expose the sample at the block face and is imaged by SEM. A section is then cut away by the microtome and the newly revealed block face is imaged. This can then be repeated multiple times to create a stack of images which can be used to reconstruct ultrastructural features in three‐dimensions. Three‐dimensional images provide a great deal more information than two‐dimensional images, as they reveal the spatial relationships between anatomical features.

The microvasculature is essential for placental function, and this study uses SBF SEM to provide new insight into its three‐dimensional structure.

## 2 METHODS

Term human placental tissue was collected after delivery from uncomplicated pregnancies with written informed consent and ethical approval from the Southampton and Southwest Hampshire Local Ethics Committee (11/SC/0529).

### 2.1 Tissue collection and fixation for electron microscopy

Villous samples from eight placentae were collected as soon as possible after delivery and small pieces (≈ 2 mm^3^) were fixed in 3% glutaraldehyde in .1 M cacodylate buffer at pH 7.4 at room temperature (RT) for 20 min and then stored at 4°C for at least 24 hr until processing for either SBF SEM or transmission electron microscopy (TEM).

### 2.2 TEM processing and imaging

Fixed placental fragments were washed twice for 10 min in .1 M sodium cacodylate buffer (Agar Scientific) at pH 7.4 containing .23 M sucrose (Fisher) and 2 mM CaCl_2_ (Fisher). The specimens were placed in 2% osmium tetroxide (Oxkem) in .1 M sodium cacodylate (Agar Scientific) at pH 7.4 for 60 min, then washed three times for 10 min with distilled water. Samples were treated with 2% aqueous uranyl acetate (Agar Scientific) for 20 min and then dehydrated using a graded ethanol series. Specimens were then treated with 50:50 Agar low viscosity (ALV) resin:acetonitrile (Fisher) overnight and infiltrated with fresh ALV resin (Agar Scientific) for 6 hr. Finally, specimens were embedded in fresh ALV resin and polymerised for 16 hr at 60°C. Gold/silver ultrathin sections were cut, stained with Reynolds lead citrate and viewed by TEM (Tecnai 12, ThermoFisher) to study interendothelial protrusion (IEP) profiles at higher resolution.

### 2.3 SBF SEM processing and imaging

Serial block‐face scanning electron microscopy is a high‐resolution technique where serial images are generated from a resin block which is sliced sequentially by an automated ultramicrotome in the chamber of the SEM. Fixed samples for SBF SEM were processed according to an established protocol (Deerinck *et al.*, 2010; Palaiologou *et al.*, 2020). Briefly, samples were washed in .1 M sodium cacodylate buffer, immersed in 2% osmium and 1.5% potassium ferrocyanide in cacodylate buffer for 60 min on ice and washed with distilled water. Samples were then immersed in a 1% aqueous solution of thiocarbohydrazide (Acros Organics) at RT for 20 min and washed with distilled water. Samples were immersed in 2% OsO_4_ (Oxkem) for 30 min at RT and washed with distilled water followed by 2% aqueous uranyl acetate at 4°C for 60 min, washed with distilled water and then in Walton's lead aspartate at 60°C for 30 min and washed with distilled water. The samples were dehydrated through a graded ethanol series (Fisher). Finally, samples were placed in acetonitrile for 20 min and 50:50 acetonitrile and ALV resin overnight. Samples were infiltrated with fresh ALV resin for 6 hr embedded and polymerized at 60°C for 16 hr. Polymerised blocks were trimmed to < 1 mm^2^, mounted on an aluminium pin with conductive glue and sputter‐coated with gold palladium.

Blocks were imaged using a Gatan 3View (Gatan) inside an FEI Quanta 250 FEGSEM (ThermoFisher) at 3.0 kV accelerating voltage, spot size 3 and with a vacuum level of 40 Pa. Stacks of images were collected at pixel sizes ranging from 2.6 to 14 nm, slice thickness from 25 to 50 nm, and the number of images per stack from 300 to 2,000.

### 2.4 Image processing and segmentation

Twenty‐seven SBF SEM image stacks of terminal villi from eight placentae were processed in fiji (version 2.0.0‐rc‐43; Schindelin *et al.*, 2012) using a Gaussian blur filter (sigma radius 2) and enhanced contrast (.4% saturated pixels). Selected regions were manually segmented in amira 6.3 (ThermoFisher). TEM images were all adjusted for brightness and contrast.

### 2.5 Quantification of pericyte coverage

To study the extent to which pericytes covered the fetal capillaries in terminal villi, the lines on the randomly offset 4 × 4 grids applied in fiji every 50 serial images were used to represent random paths. Using the horizontal lines, the number of times the lines intersected the capillary basal membrane and then the pericyte, was determined and expressed as a ratio of pericyte/capillary intersects.

### 2.6 Quantification of interendothelial protrusions and regions of complex endothelial junction

Interendothelial protrusions occupy too small a volume to allow practical quantification using stereological approaches. The IEPs were therefore identified visually and marked using a region of interest manager to avoid double counting. IEPs were identified by one investigator (E.P.), with discussion with R.L. IEPs appeared as discrete vesicles within the cytoplasm of an endothelial cell which, when viewed in multiple consecutive images, formed a continuous structure back to the endothelial junction with an adjacent endothelial cell.

The IEPs, which were long and thin protrusions into the neighbouring cells, were distinguished from regions of complex endothelial junction, which were wider and relatively shallow indentations into neighbouring cells.

The spatial coordinates for the tip and the base of each IEP were determined within the stack, allowing the length of a straight line between these points to be calculated. This measure was used as an estimate and will tend to underestimate length where IEPs are not straight, but it was not practical manually to segment all the IEPs.

The width of the IEPs was measured at the widest point in the IEP (not including the initial section where it initially forms from the parent cell).

To estimate the density of IEPs, the total number of IEPs was counted and expressed in relation to the total villous volume of tissue and scaled up to 1 cm^3^. This value was further adjusted to take into account the volume of the intervillous space (34% of placental volume; Mayhew, 2009) where there are no IEPs. Finally, as the IEPs were only counted in terminal villi, we further adjusted the value for the volume percentage of terminal villi (38.7% of villous volume; Sen *et al.*, 1979).

### 2.7 Counting endothelial junctions in capillary profiles

To estimate the number of endothelial junctions in villous capillaries, full capillary cross‐sectional profiles were identified in each of the images used for stereology in the 27 terminal villous stacks. Regions above and below the capillary profile were inspected to ensure that it was a full cross‐section and that the profile was not created by taking the edge off one side of a capillary. Capillary diameter was estimated in full capillary profiles by measuring at the widest point.

### 2.8 Counting endothelial junctions and measuring diameter in the arteriole and venule

To estimate the number of endothelial junctions in the arteriole and venule in the intermediate villi stack, these were counted in the arteriole and venule cross‐sections in every 25th slice. Arteriole and venule diameters were determined by measuring the widest point every 25 slices and averaging this. For these purposes, we defined the arteriole as the smaller higher resistance vessel and the venule as the larger lower resistance vessel.

### 2.9 Data analysis and statistics

For stereological analysis, running means were plotted to assess the adequacy of the sample size. Comparisons between groups were made using Student's *t*‐test in SPSS. As capillary diameter data were not normally distributed, correlations were performed using Spearman's rho in IBM SPSS^®^ Statistics 24. Data are presented as mean and SD or median and range as appropriate.

## 3 RESULTS

This study included eight term placentas from uncomplicated pregnancies, five collected following vaginal delivery and three following elective caesarean sections with an average gestational age was 283 ± 8 days (40 weeks and 2 days).

### 3.1 Capillary segmentation

**FIGURE 1:**
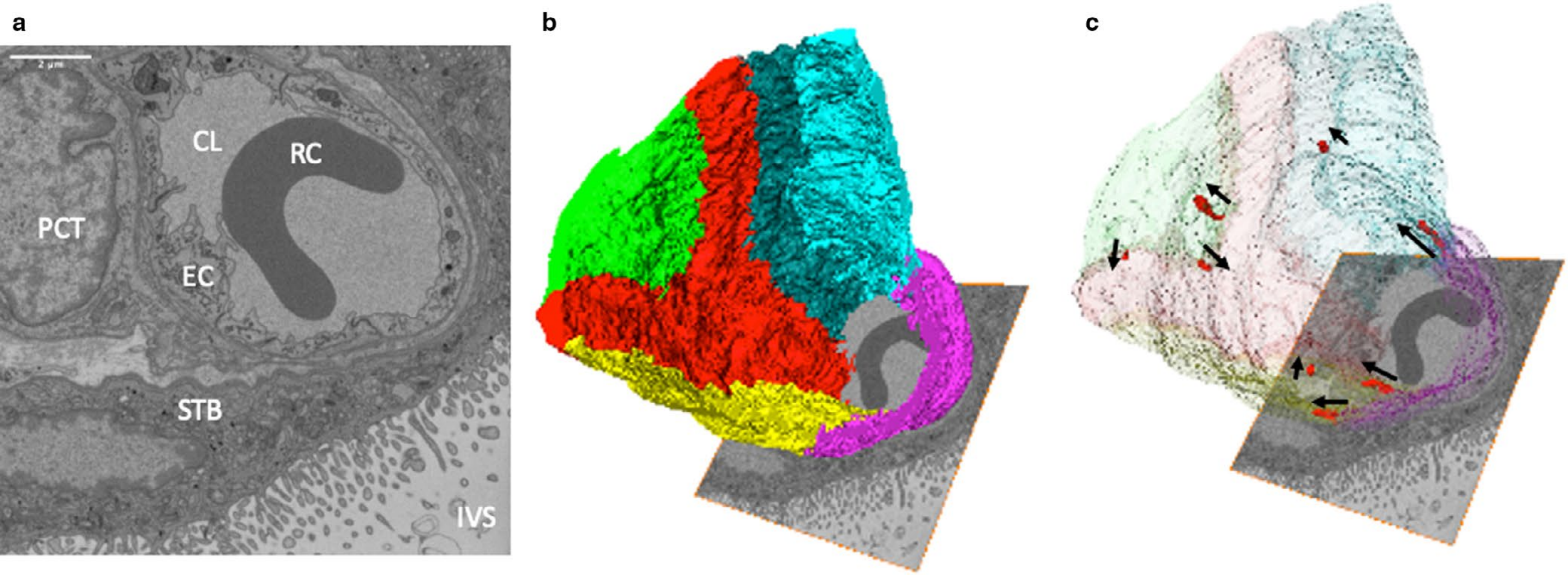
Three‐dimensional reconstruction of a region of capillary from a terminal villus. (a) One of the 367 serial images in the SBF SEM stack used to reconstruct the capillary in three dimensions (12.6 × 12.6 × 18.35 μm). The image shows the capillary lumen (CL) containing a red blood cell (RC), the surrounding endothelial cells (EC), a pericyte (PCT), the syncytiotrophoblast (STB) and the intervillous space (IVS). Additional images from the stack can be seen in Appendix S1. (b) A three‐dimensional reconstruction of the stack showing the organisation of different endothelial cells, each of which is presented in a different colour. (c) The same image with the endothelial cells made transparent to reveal the location of IEPs (red) with their orientation highlighted by arrows pointing in the direction in which they protrude

**FIGURE 2:**
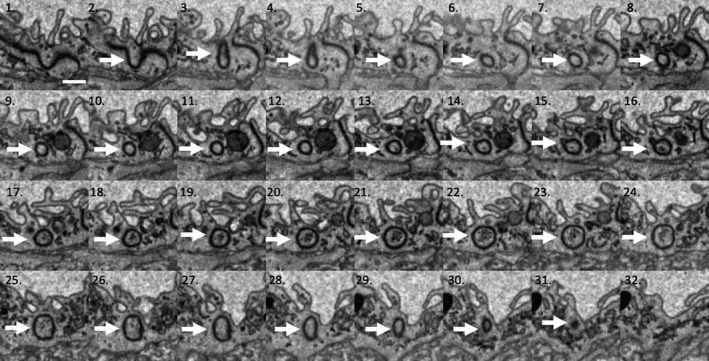
A set of SBF SEM serial images from the series stack reconstructed in Figure 1. This shows an IEP beginning as a fold in the cell–cell junction in image 1 and disappearing in image 32. All images are 50 nm apart, so the overall length of the IEP is approximately 1.5 μm. The IEP is identified by an arrow sitting within the acceptor endothelial cell. The capillary lumen is at the top of each picture. Scale bar: (image 1) .5 μm

**FIGURE 3:**
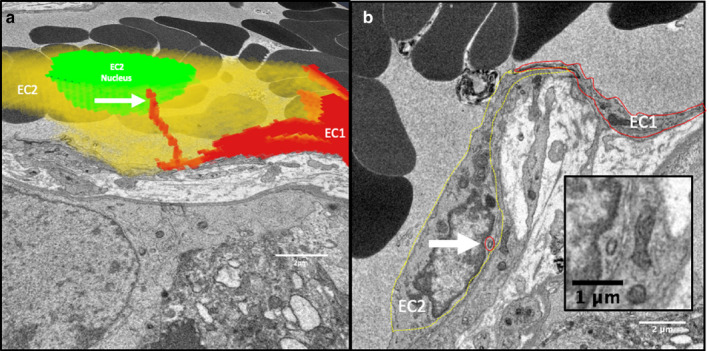
IEPs extend from one endothelial cell into another. (a) A three‐dimensional reconstruction of two partial endothelial cells (red [EC1] and yellow [EC2]) based on 80 serial images 50 nm apart. An IEP from EC1 extends within EC2. (b) A single section from the image stack showing EC1 (solid red line) and EC2 (dashed yellow line) with the cross‐section of the IEP from EC1 within EC2 highlighted by a red circle. The inset shows a higher magnification image of the IEP cross‐section

An 18.35‐μm‐long section of capillary from a terminal villus containing seven partial endothelial cells was segmented (Figure 1). This image sequence shows the transition between dilated and non‐dilated terminal villus capillary. No endothelial cell nuclei were included in the imaged region. A notable feature revealed by this segmentation was the presence of eight IEPs, each extending from the lateral membrane of one endothelial cell inside the neighbouring recipient endothelial cell (Figure 1c). The plasma membrane of the recipient cell closely surrounded each IEP, with both cells maintaining the integrity of their respective plasma membranes (Figures 2 and 3).

### 3.2 Interendothelial protrusions

Interendothelial protrusions were observed in samples from all eight placentae studied. In the 27 SBF SEM stacks, a total of 89 IEPs were identified within all the capillaries therein. The median (range) length of the IEPs (measured as a straight line from tip to base) when calculated per placenta, was 1.74 (.2–5.2) μm and the width was .23 (.15–.50) μm (*n* = 8). The median IEP length to width ratio was 9.7 (4.2–34.8). As not all placentae were equally represented, for comparison the overall median length was 1.6 (.4–7.1) μm (*n* = 88; one IEP length could not be determined due to a jump in the stack) and the overall width was .21 (.06–.52) μm (*n* = 88). In two cases, branched IEPs were observed. In both cases, the second branch of the IEP was much smaller than the first (Figure 3).

There were 89 IEPs observed in 6.41 × 10^14^ nm^3^ of terminal villous tissue, which equates to 139 million IEP per cm^3^ of terminal villous tissue. However, this figure needs to be adjusted to account for the intervillous space (35% of placental volume), which will contain no IEPs, and the fact that terminal villi constitute 39% of villous volume. Accounting for these factors, this gives an estimated 35 million IEPs in terminal villi per cm^3^ of villous tissue.

### 3.3 IEP profiles

**FIGURE 4:**
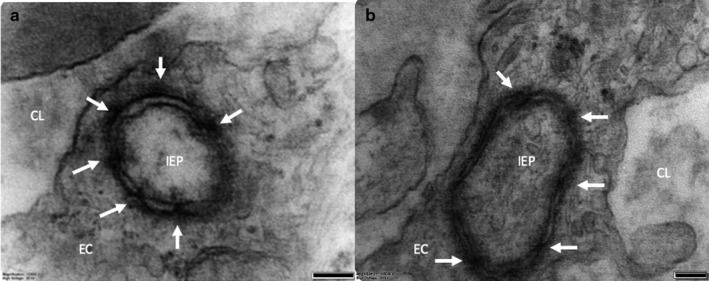
TEM images showing probable IEP cross‐sections in two different placentae (a and b). Junctional complexes are indicated by the white arrows connecting the IEP and the recipient cell. In (a), the membranes on the lower right‐hand quadrant of the IEP are unclear, which is likely due to the membranes not being perpendicular to the plane of the sample and thus not distinguishable. CL, endothelial lumen; EC, endothelial cell; IEP, intracytoplasmic process. Scale bars: 100 nm

Although it is not possible definitively to identify IEPs observed in two‐dimensional sections, inspection of TEM sections for structures consistent with cross‐sections of IEPs identified likely structures in four different placentae (Figure 4). These small structures had inner and outer membrane rings, representing the recipient and donor cells, respectively, with junctional complexes linking the membranes of the two cells (Figure 4).

### 3.4 IEPs in arterioles and venules

Few intermediate villous stacks were available and these were of lower magnification, so it was not possible to characterise IEPs in vessels from this type of villus. However, one IEP was observed in an arteriole.

### 3.5 Complex endothelial junctions

In addition to the IEPs, 133 regions of complex endothelial junction were identified. The median (range) depth of these regions was .47 (.10−2.73) μm and width of .20 (.06–.65) μm.

### 3.6 Endothelial junctions in capillary cross‐sections

**FIGURE 5:**
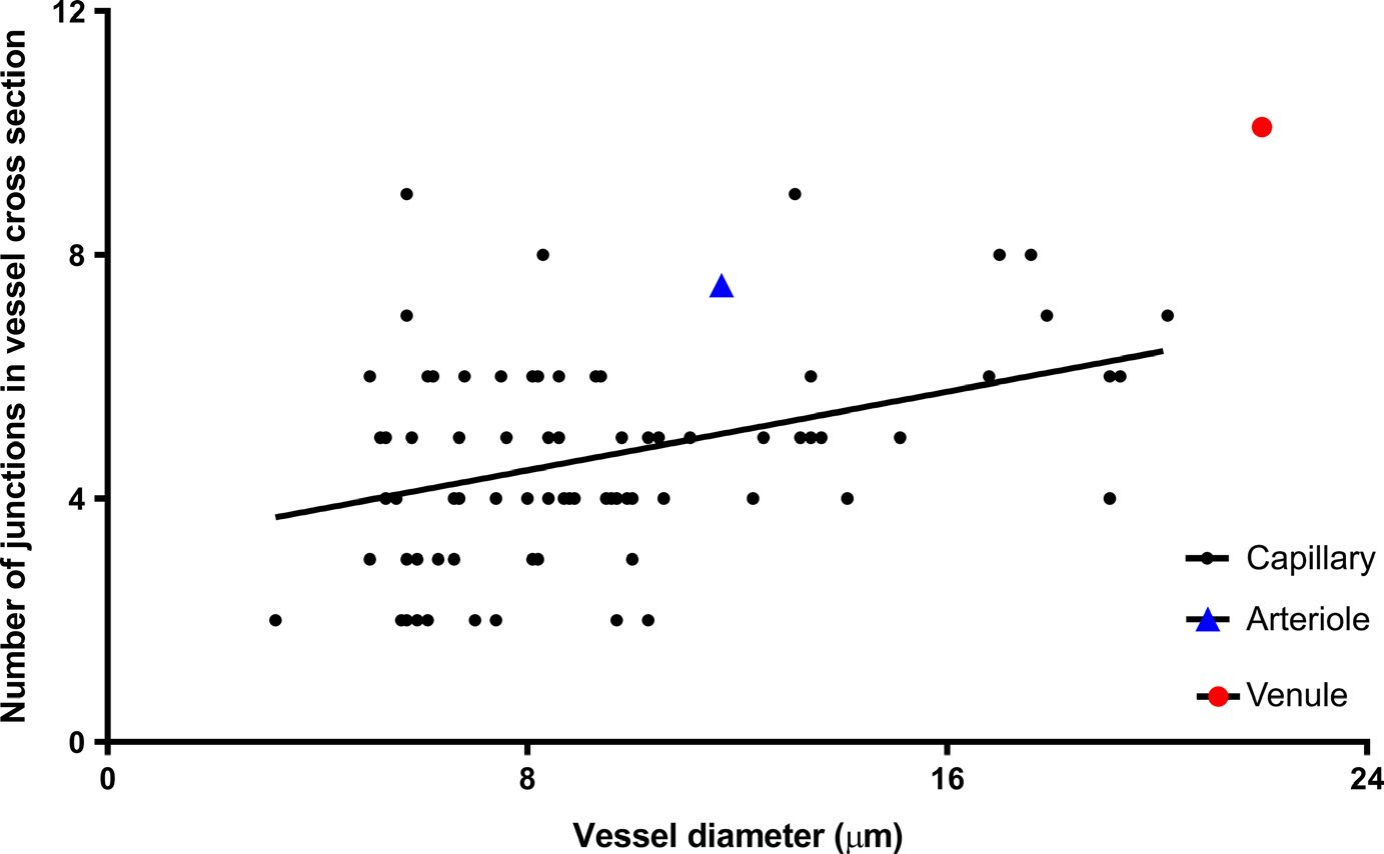
The number of endothelial junctions is related to the capillary diameter. *ρ* = .31, *p* = .007, *n* = 77 capillary profiles. For comparison, the data for the segment of arteriole and venule are also plotted; the data are not included in the regression analysis

The average number of junctions per capillary was 4.5 ± 1.4 (*n* = 13 capillaries, from eight placentae). Within terminal villi, sections of the capillary closely associated with the trophoblast basal lamina are often dilated, and nutrient and gas exchange is thought to be more efficient in these regions due to low diffusion distance. The number of junctions in non‐dilated capillary profiles (4.3 ± 1.6, *n* = 58, defined as <11 μm wide) was significantly lower than the average number of junctions in dilated regions of capillaries (5.8 ± 1.4, *n* = 19, defined as >11 μm; *p* < .001). The number of junctions/capillary profile was positively related to the cross‐sectional capillary diameter (*ρ *= .31, *p* = .007, *n* = 77; Figure 5).

### 3.7 Putative trans‐endothelial channel

**FIGURE 6:**
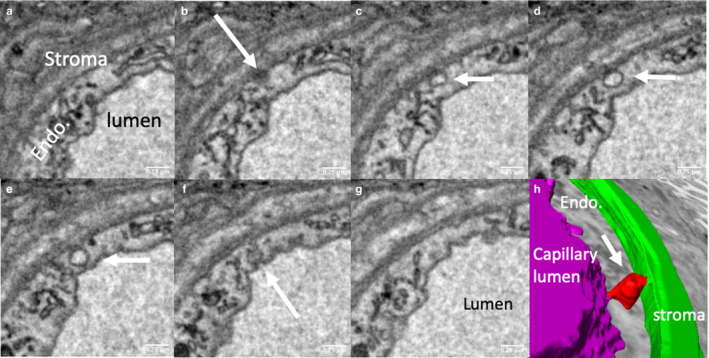
Serial sections illustrating a putative trans‐endothelial channel. The trans‐endothelial channel indicated by white arrows in (b–f) opens on the abluminal side in (b) and on the luminal side in (f). (h) A three‐dimensional reconstruction of the channel (red), connecting the lumen (purple) and the villous stroma (green). The reticular structures indicated by black arrows in (c and g) appear to connect the luminal and abluminal surfaces; it is conceivable that these could be adapted to form channels

A structure consistent with a trans‐endothelial channel was observed in the three‐dimensional stacks (Figure 6). Reticular structures could also be seen in contact with both the apical and basal side of the endothelial cell but there was no evidence that these opened into the lumen or stroma.

### 3.8 Arteriole and venule

**FIGURE 7:**
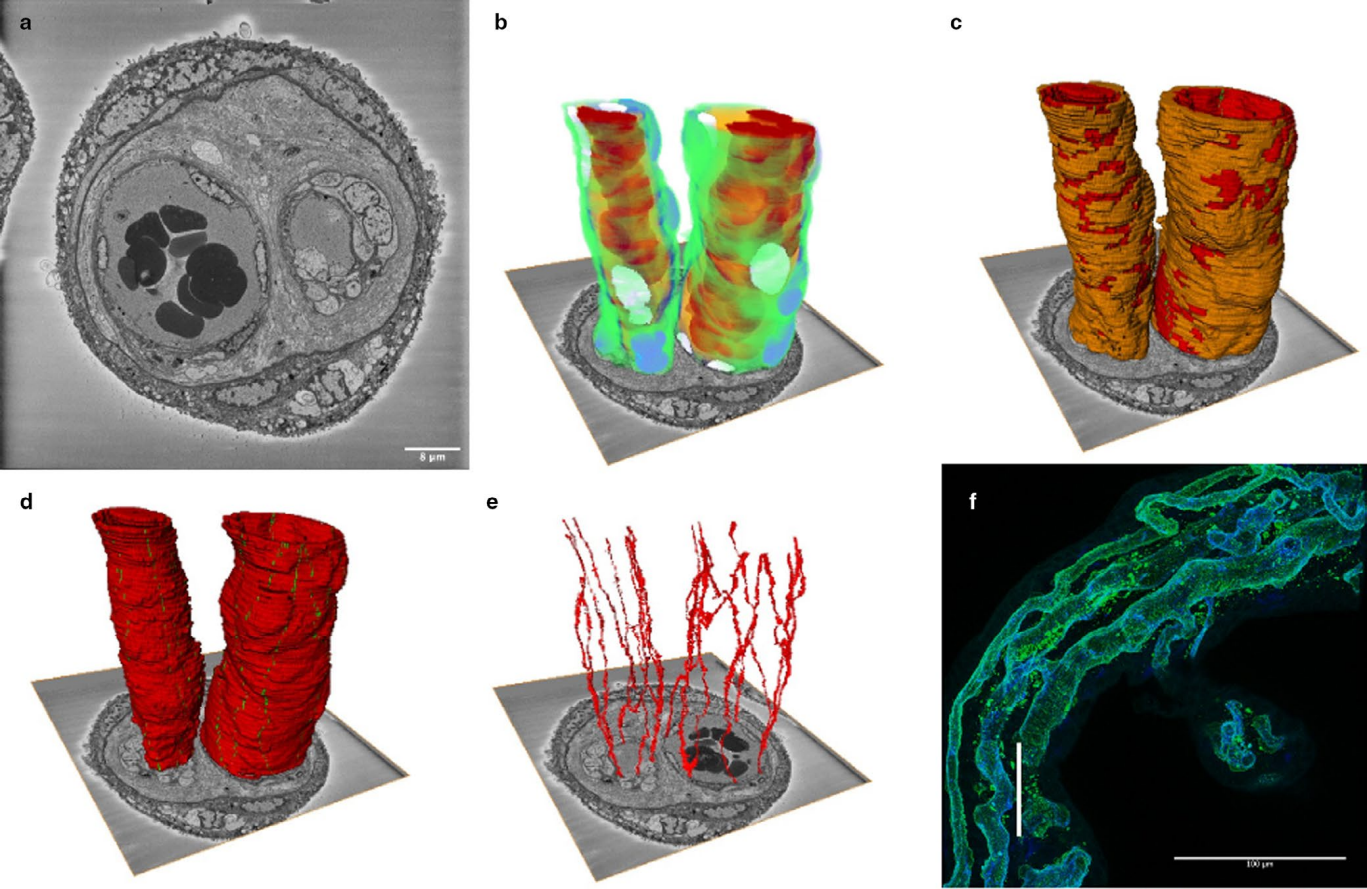
Three‐dimensional reconstruction of an arteriole and venule from human term placenta. This image is based on 540 serial images (100 nm apart) from an arteriole and a venule pair from an intermediate villus. We suggest that the larger, lower resistance vessel on the right is the venule and the smaller vessel on the left the arteriole. (a) A representative image from the SBF SEM image stack. (b) A reconstruction of the arteriole and venule showing pericytes in green, pericyte nuclei in purple, endothelial cells in blue, endothelial nuclei in white, plasma in yellow and erythrocytes in red. (c) The arteriole and venule showing that pericytes (orange) cover a high proportion of both vessels. Endothelial cells are shown in red and the junctions in green. (d) The two vessels showing the endothelial cells in red and the junctions in green. (e) The endothelial cell junctions of the arteriole and venule. The difference in the arrangement of endothelial cells is most clearly seen when this image is rotated, and a movie is provided in Appendix S1. (f) To provide context and scale, this confocal image shows a similar region of intermediate villus with the vertical white scale bar representing the size of the SBF SEM stack (54 μm). The endothelium is blue and the pericytes green. There are three vessels in this villus; note the anastomosis between two of these vessels

In a 54‐μm‐long section of intermediate villus, the two parallel vessels were segmented to show the endothelial cells, endothelial junctions, pericytes, plasma and erythrocytes (Figure 7). Digital micrograph software (Gatan Inc.) was used to correct for drift within the arteriole and venule stack. The larger vessel (believed to be the venule) had an average diameter along its length of 22.0 ± 1.3 μm compared with a smaller vessel (believed to be the arteriole) with an average diameter of 11.7 ± 1.8 μm (*p* < .001, *n* = 22 observations). On average, cross‐sections of the larger vessel had 10.1 ± .9 junctions along the length vs. 7.5 ± 1.4 for smaller vessels (*p* < .001, *n* = 22).

## 4 DISCUSSION

This study investigates the ultrastructure of the placental microvasculature in three dimensions. A key finding was the identification of a novel intercellular connection between endothelial cells, the IEPs. This study also provided insight into the arrangement of endothelial cells and structures that may influence capillary permeability.

The IEPs are thin projections from one endothelial cell that lie completely enclosed within the cytoplasm of the neighbouring endothelial cell. The IEPs are an ultrastructural feature which would be very difficult to visualise without multiple serial sections and we are not aware of similar structures reported in the literature. Regions of complex endothelial junctions have been described, and we also see these in the placenta, but we regard the IEPs as structurally distinct. However, it is possible that in a dynamic junctional region, IEPs and complex junctions represent different stages of the same process. Alternatively, complex junctions and IEPs may be distinct but related phenomena in the way that lamellipodia and filopodia are related (Small *et al.*, 2002). Future studies need to address the mechanisms underlying these structures and their function.

Microvilli, filopodia, tunnelling nanotubes and cilia have similar shapes to IEPs but there are distinct differences (Mattila and Lappalainen, 2008; Fisch and Dupuis‐Williams, 2011; Li *et al.*, 2019). IEPs did not have basal bodies and no microtubules could be observed, so they are not a form of cilia or primary cilia (Fisch and Dupuis‐Williams, 2011). Tunnelling nanotubes are long, thin membranous protrusions that can be shown to connect cells in culture, but typically these connect cells that are not directly adjacent (Li *et al.*, 2019). Filopodia can also be observed at the leading edge of motile cells in culture and it is possible that the IEPs are an in vivo equivalent of filopodia. Characterisation of the proteins mediating the formation of IEPs may help determine their relationships to these previously described structures.

The role of IEPs within the endothelium is not clear but we suggest they could mediate cell–cell communication by either mechanosensing or paracrine signalling. The IEPs appear to be tightly anchored within the adjacent cell via intercellular junctional complexes which may allow them to sense mechanical stresses between cells. Receptors within the IEP junction could mediate highly targeted paracrine signalling between adjacent endothelial cells. If IEPs produced nitric oxide (as primary cilia are known to do (Saternos and AbouAlaiwi, 2018), this would be directly targeted at the recipient cell. In live tissue, the cells are likely to be dynamic, and IEPs could help coordinate movement between adjacent cells. If this is the case, they could be related to filopodia.

Transmission electron microscopy of junctional complexes in the probable IEP cross‐sections suggests the presence of tight junctions due to the presence of electron dense material within the junction. However, given the relatively limited number of examples, we cannot rule out the presence of other types of junction such as adherens junctions, which could mediate attachment to the cytoskeleton. Alternatively, if connecting gap junctions were shown to be present along the IEP, this would allow ionic and electrical cell–cell communication.

The density of IEPs was estimated to be 35 million cm^–3^ and although this estimate includes a number of assumptions, we are confident that IEPs are abundant and consider it likely that most or all terminal villous endothelial cells have them. It will be interesting to determine whether IEPs are present in other tissues; if they are, the locations in which they are found may help identify their role.

As seen in the reconstructed sections of vessels, the endothelial cells typically had pointed ends, suggesting a roughly diamond shape, although one was bifurcated, creating a Y‐shape. The cells were aligned longitudinally along the capillary, parallel with the direction of flow, and there was variation in the width of different cells.

The permeability of the placental microvasculature will be an important determinant of placental nutrient and waste transfer. The presence of multiple cells around the circumference of the capillary increases the number of endothelial junctions through which diffusion can occur. Despite the limited data on the number of endothelial cells around capillaries in different tissues, it would be interesting to determine whether the number of cells/junctions relates to the nutrient transfer requirements in other tissues.

There was a positive relationship between capillary endothelial cell number and vessel diameter, but although increasing vessel diameter did increase cell number, it did not lead to a proportionate increase in cell number. This suggests a plasticity in endothelial cell size.

Permeability of capillaries will depend on both the permeability of the endothelial junction and the number of endothelial junctions. This study suggests that in dilatated regions of capillary there tend to be more endothelial junctions, which should increase the capacity for diffusion. However, the increase in the number of junctions is not proportional to the increase in diameter of the capillary, so any impact on diffusion will depend on whether diffusion is limited by the total length of endothelial junction in a region of vessel.

Pericyte coverage has the potential to inhibit nutrient diffusion in and out of the placental vessels through endothelial junctions or transcellular channels. Most solute exchange is thought to occur in the capillaries and here pericyte coverage appeared to be much lower than observed in the segmented region of arteriole and venule. Further work is required to obtain a more representative analysis of arteriole and venule coverage. Pericyte coverage of endothelial junctions could influence paracellular diffusion.

The existence of trans‐endothelial channels could have a profound influence on the permeability of placental capillaries. The clearest example was of a large vesicle‐shaped channel in close contact with the basal side of the endothelium with a clear opening to the luminal side. However, only one clear example of this was observed and it is not clear how these trans‐endothelial channels form. One speculative mechanism would be via adaptation of reticular structures observed to be in direct contact with both the apical and basal plasma membranes of the endothelial cell. The role of trans‐endothelial channels in the placental capillary endothelium requires further investigation.

Serial block‐face scanning electron microscopy imaging of a paired arteriole and venule demonstrates the endothelial cell architecture and pericyte coverage in the two vessels. We suggest that the smaller, higher resistance vessel, with thicker walls along its length, is the arteriole and the larger vessel is the venule. It has previously been reported that venules in mature intermediate villi are smaller than arterioles but we do not think that this can be the case in intermediate villi shown here, as the resistance within the venous circulation would then be considerable (Kaufmann *et al.*, 1985).

This study allows us to view the three‐dimensional structure of the placental microvasculature in unprecedented details. However, current limitations of SBF SEM are the cost of producing the stacks and the time required to segment anatomical features within these stacks. Automated segmentation is required to take full advantage of these datasets and machine learning‐based approaches may make this possible in the future (Hesamian *et al.*, 2019).

The IEPs described in this study represent a novel intercellular connection. Although the function of the IEPs remains unclear, we hypothesise that they are involved in mechanosensing between adjacent endothelial cells and in the maintenance of endothelial integrity. It now needs to be determined whether IEPs are present in capillaries from other tissues and how they are affected in disease states.

## Supporting information

Figure S1

Video S1

## Data Availability

The data that support the findings of this study are available from the corresponding author upon reasonable request.
