# Supplementary material for: Serial block‐face scanning electron microscopy reveals novel intercellular connections in human term placental microvasculature
Source: J Anat. 2020 Apr 3;237(2):241–9. doi: 10.1111/joa.13191 (PMC7369196; doi:10.1111/joa.13191)
Supplement: Supplementary file 1 — Figure S1 [file JOA-237-241-s001.docx]

**Supplementary material**

For figure 1: additional images from the stack segmented in figure 1 showing different depths (slice 49, 133, 246 and 311). IEPs can be seen in some images indicated by white arrows.


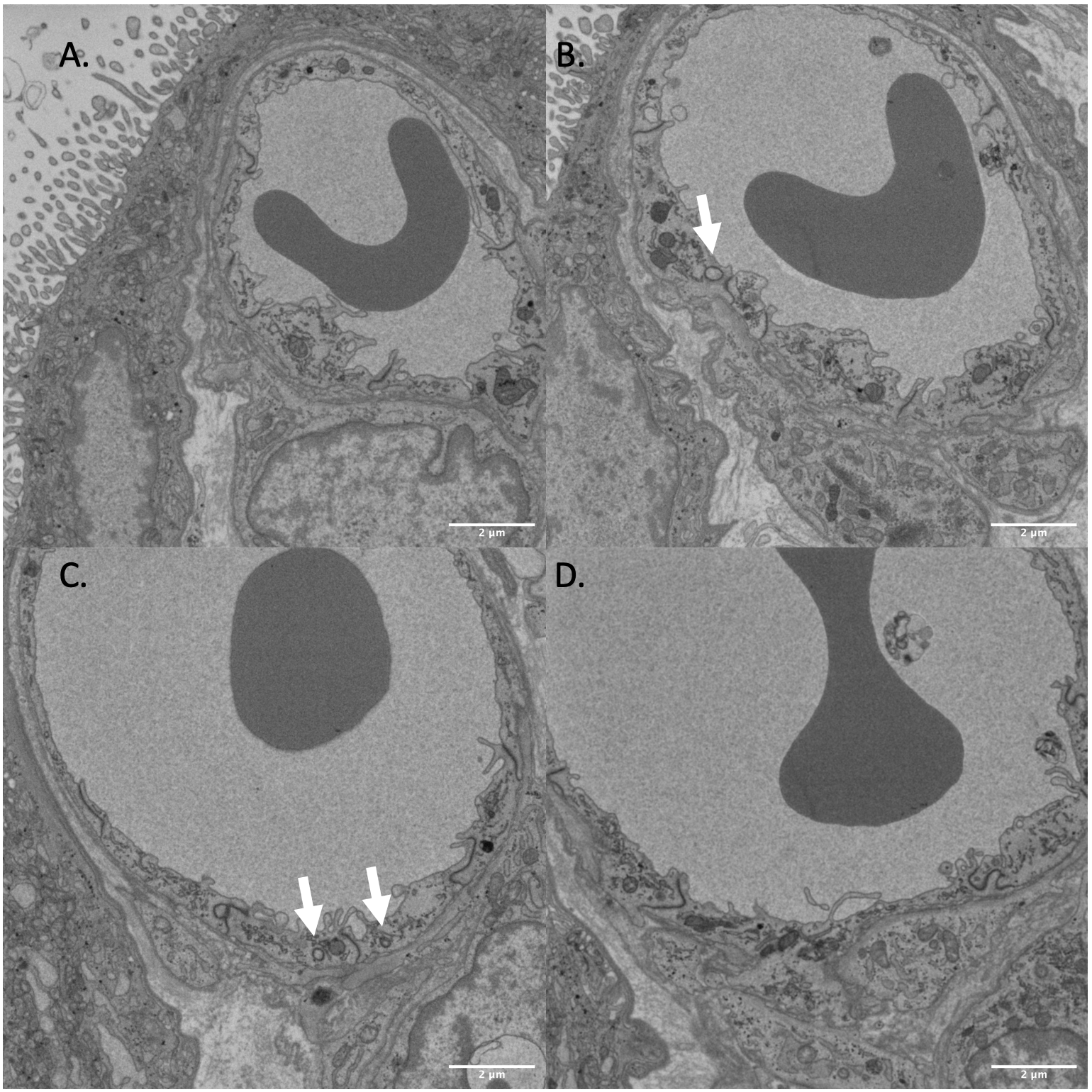


Supplementary video to support figure 7: this video shows the arteriole (blue endothelial junctions) and venule (red endothelial junctions) with the endothelial cells in transparent yellow. The outlines of the nuclei can be seen within some endothelial cells.
